# Bacterial bioburden and community structure of potable water used in the International Space Station

**DOI:** 10.1038/s41598-022-19320-3

**Published:** 2022-09-29

**Authors:** Tomoaki Ichijo, Kimiko Uchii, Kazuma Sekimoto, Takashi Minakami, Takashi Sugita, Masao Nasu, Takashi Yamazaki

**Affiliations:** 1 Faculty of Health and Nutrition, Osaka Shoin Women’s University, Higashi-Osaka, Japan; 2 Graduate School of Human Sciences, Osaka Shoin Women’s University, Higashi-Osaka, Japan; 3 Faculty of Pharmacy, Osaka Ohtani University, Tondabayashi, Japan; 4 Particle Counter Division, RION Co., Ltd., Kokubunji, Japan; 5 Department of Microbiology, Meiji Pharmaceutical University, Kiyose, Japan; 6 Graduate School of Pharmaceutical Sciences, Osaka Ohtani University, Tondabayashi, Japan; 7 General Medical Education and Research Center, Teikyo University, Tokyo, Japan; 8 JEM Utilization Center, Human Spaceflight Technology Directorate, Japan Aerospace Exploration Agency (JAXA), Tsukuba, Japan

**Keywords:** Environmental microbiology, Water microbiology

## Abstract

The control of microbes in manned spaceflight is essential to reducing the risk of infection and maintaining crew health. The primary issue is ensuring the safety of a potable water system, where simultaneous monitoring of microbial abundance and community structure is needed. In this paper, we develop a flow cytometry-based counting protocol targeting cellular flavin autofluorescence as a tool for rapid monitoring of bacterial cells in water. This was successfully applied to estimate the bacterial bioburden in the potable water collected from the International Space Station. We also demonstrate the efficacy of the MinION nanopore sequencer in rapidly characterizing bacterial community structure and identifying the dominant species. These monitoring protocols' rapidity and cost effectiveness would contribute to developing sustainable real-time surveillance of potable water in spaceflight.

## Introduction

The control of microbes is essential to reduce the risk of infection and ensure crew health in long-duration spaceflight. As a primary step to achieving this goal, microbial communities inside the International Space Station (ISS) have been periodically monitored, i.e., surfaces, air, and water. The dynamics of bacterial community structure on surfaces inside the ISS revealed that microbial communities in the ISS environment were mostly human-associated as represented by the families *Staphylococcaceae* and *Enterobacteriaceae,* which are predominant members of the skin and gut microflora​​, respectively^1,2^. The bacterial community in the air was also likely to associate with humans, although its community structure differed from surfaces^3^. In contrast, drinking water supplied from the Potable Water Dispenser (PWD) was characterized by bacterial species commonly found in potable water systems on the ground and were considered to be introduced before the launch of PWD^4–6^. Since these contaminants included opportunistic bacterial pathogens, such as *Burkholderia* spp. and *Ralstonia* spp., which can cause infections in humans with a weak immune system, health concerns arise about the safety of potable water.

In the ISS, water samples are collected and returned to the ground on a quarterly basis and monitored by culture methods. Requirements for potable water supplied from the PWD are defined as maximum 50 CFU/mL for bacterial and not detectable for coliform contaminants^7,8^ and maximum total organic carbon (TOC) limit of 3 mg/L measured by the onboard TOC analyzer (TOCA)^9^, respectively. However, culture-based colony forming unit (CFU) method is time-consuming and TOC is not a direct measure of microbial contaminants. As to the bacterial diversity, bacteria in the potable water of the ISS have been analyzed using culture methods, and *Ralstonia* spp., *Sphingomonas* spp., and *Pseudomonas* spp. have been isolated, as well as opportunistic pathogens such as *Stenotrophomonas maltophilia* and *Pseudomonas aeruginosa*^7,10^. It is also known that bacterial CFU counts in the water of the ISS have exceeded acceptable limits several times in previous monitoring^11^. To control the bacterial bioburden in the PWD water, continuous real-time monitoring of the abundance and structure of bacterial communities is desirable since it would enable rapid alert and action based on changes in the bioburden and bacterial community structure before the crew is exposed to bacterial risks. In the monitoring of bioburden, the use of a biofluorescent particle counter based on flow cytometry is promising because it can rapidly quantify the number of bacteria. Furthermore, a protocol that detects bacterial cells without staining has excellent speed and would be easily automated, utilizing the autofluorescence of bacterial cells. In monitoring bacterial community structures, a portable nanopore sequencer (Oxford Nanopore Technologies, ONT) is a good candidate. It provides rapid real-time sequencing by monitoring electrical signals when DNA molecules pass through protein nanopores^12,13^. The smallest ONT device, the MinION, has already been tested aboard the ISS using mock microbial communities, exhibiting high potential for onboard use^14,15^.

Our study demonstrates the application of a biofluorescent particle counter protocol that directly enumerates bacterial cells and the MinION real-time sequencing platform, which rapidly clarifies bacterial community structures, for monitoring the bacterial bioburden in PWD water. The accuracy of the biofluorescent particle counter protocol was assessed by cell counting under fluorescence microscopy, and that of the MinION was evaluated by comparing it with the current preferred Illumina sequencing platform. The results of this study are expected to contribute to the development of a real-time surveillance system of potable water for spaceflight.

## Results

### Total direct count and CFU count in the PWD water

Bacteria present in the PWD water were counted by direct microscopic and plate count methods. The total number of bacteria was 8.2 × 10^4^ cells/mL by 4′,6-diamidino-2-phenylindole (DAPI) staining, and that counted by 6-carboxyfluorescein diacetate (6-CFDA) staining, which uses esterase activity as an indicator, was 7.8 × 10^4^ cells/mL. Colony count with R2A agar and tryptic soy agar (TSA) agar were 4.4 × 10^4^ CFU/mL and 2.4 × 10^4^ CFU/mL, respectively. These results indicated that many of the bacteria were physiologically active and culturable both on low and universal nutrient media.

### MALDI TOF–MS analysis for identification of bacterial isolates

Bacterial isolates were identified by a matrix-assisted laser desorption/ionization time-of-flight mass spectrometry (MALDI TOF–MS) analysis. All 18 isolates from TSA and 19 out of 20 isolates from R2A were identified as *Ralstonia pickettii* (Supplementary Table 1). 16 isolates from TSA and 15 isolates from R2A had score values ≧ 2.3, which secures highly probable species identification. 2 isolates from TSA and 4 isolates from R2A had score values ≧ 2.0, which secures genus identification and probable species identification. One isolate from R2A was not identified.

### Protocol setup for bacterial cell count by a biofluorescent particle counter

**Figure 1:**
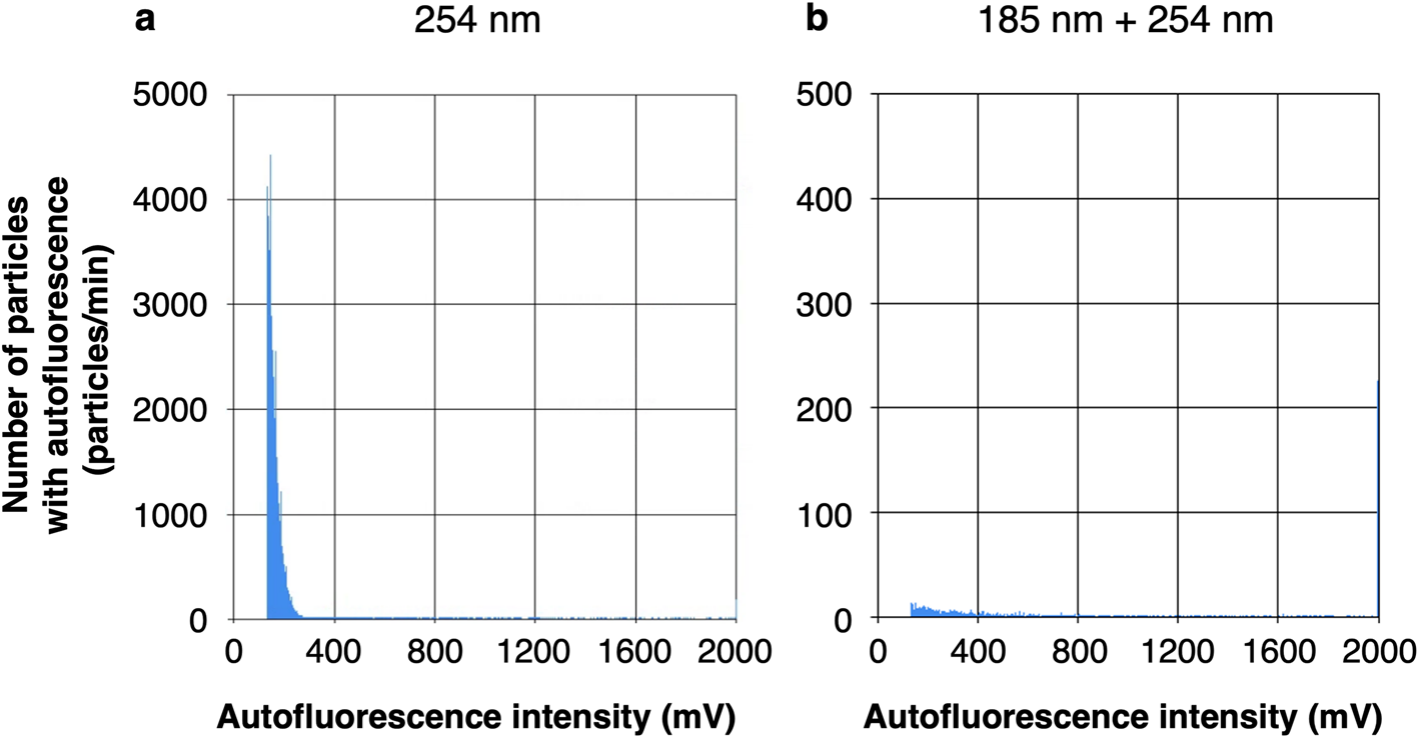
Particle count with autofluorescence in the PWD water passed through a 0.2 µm filter by a biofluorescent particle counter with deep UV irradiation at 254 nm (**a**) and 185 + 254 nm (**b**). The total numbers of particles counted as autofluorescent particles were the cumulative total of the number of particles on the vertical axis from 133 to 1200 mV on the horizontal axis, 1.5 × 10^4^ particles/mL (**a**) and 1.6 × 10^2^ particles/mL (**b**).

Bacterial cell counting protocol that detects autofluorescence of bacterial cells was developed using a commercially available biofluorescent particle counter (XL-10BT1, Rion Co. Ltd.). Since potable water often contains dissolved organic carbon that emits autofluorescence and may increase the background, it is very important to remove dissolved organic carbon before measurement. Therefore, to verify the effectiveness of deep UV irradiation at a wavelength of 254 nm or two wavelengths of 185 nm and 254 nm in decomposing dissolved organic carbon, the PWD water was passed through a 0.2 µm filter to remove bacterial cells but retain dissolved organic carbon and measured with or without deep UV irradiation. In these histograms showing the distribution of particles (Fig. 1), the number determined and counted as autofluorescent particles by the biofluorescent particle counter was 1.5 × 10^4^ particles/mL after deep UV irradiation at 254 nm in the PWD water passed through a 0.2 µm filter (Fig. 1a). On the other hand, it was reduced by about 99% to 1.6 × 10^2^ particles/mL by deep UV irradiation at both 185 nm and 254 nm (Fig. 1b). It was suggested that the autofluorescence signals, which were counted as autofluorescent particles, originated from dissolved organic carbon and disappeared due to their decomposition under 185 + 254 nm irradiation. Based on these results, the wavelength of the deep UV irradiation device upstream of the biofluorescent particle counter was set to 185 + 254 nm for the bacterial cell counting of the PWD water.

In the filtered PWD water, some autofluorescence signals were still observed after decomposition of dissolved organic carbon by 185 + 254 nm irradiation. To confirm that these signals were originated from the ISS Potable Water Sampling Bag, the number of particles in the ground control water (ultrapure water stored in the same bag, see Materials and Methods for details) was measured. The number of autofluorescent particles of the ground control water was 1.9 × 10^2^ particles/mL, which was almost the same as that of the PWD water passed through a 0.2 µm filter after the 185 + 254 nm irradiation, and the histogram showed the similar distribution of particles (Fig. 1b). In addition, autofluorescent particles observed in the ground control ultrapure water before it was sealed in the ISS Potable Water Sampling Bag was less than 10. These results suggest that the dissolved organic carbon originated from the PWD was almost completely decomposed by 185 + 254 nm deep UV irradiation and almost all components counted as autofluorescent particles in the filtered PWD water after 185 + 254 nm deep UV irradiation were those originated from the ISS Potable Water Sampling Bag (see Fig. 1b and Supplementary Fig. 1).

**Figure 2:**
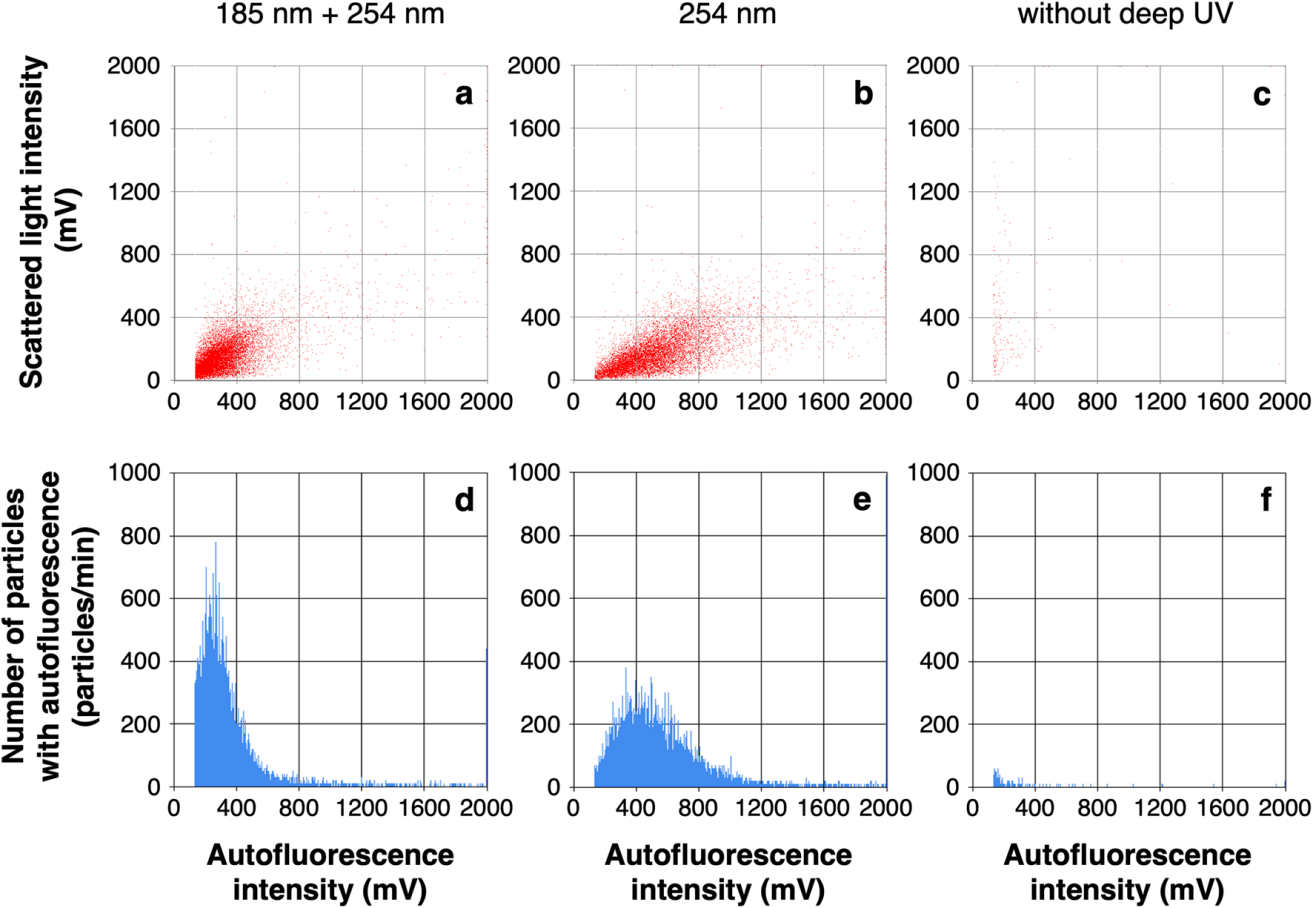
Bacterial count in the PWD water after 1:100 dilution by a biofluorescent particle counter with deep UV irradiation at 185 + 254 nm (**a**, **d**), at 254 nm (**b**, **e**) and without deep UV irradiation (**c**, **f**). Horizontal axis indicate the intracellular flavin-derived autofluorescence intensity of the measured particles (**a**–**c**). The histograms (**d**–**f**) show the number of particles with autofluorescence intensity exceeding 133 mV on the vertical axis. The total numbers of particles counted as autofluorescent particles were the cumulative total of the number of particles on the vertical axis from 133 to 1200 mV on the horizontal axis, 1.1 × 10^5^ particles/mL (**d**), 1.1 × 10^5^ particles/mL (**e**), and 1.7 × 10^3^ cell/mL (**f**).

While deep UV irradiation has been shown to be effective in removing dissolved organic carbon, it is necessary to clarify whether the difference in irradiation, i.e., 254 nm only and 185 + 254 nm, affects intracellular flavin oxidation and bacterial cell counting results. Then, to verify the effectiveness of deep UV irradiation in oxidizing intracellular flavin and enhancing autofluorescence intensity, bacterial cells were measured by a biofluorescent particle counter after deep UV irradiation at both 185 nm and 254 nm, only 254 nm and without deep UV irradiation (Fig. 2). To minimize signals derived from dissolved organic carbon that cannot be decomposed without deep UV irradiation, the PWD water was diluted 1:100 with ultrapure water and measured after irradiation at both 185 + 254 nm, only 254 nm and without deep UV irradiation. Figures 2a–c show the flavin-derived autofluorescence intensity of the measured particles on the horizontal axis and the scattered light intensity on the vertical axis. Figures 2d–f show the number of particles with autofluorescence intensity exceeding 133 mV on the vertical axis and the autofluorescence intensity on the horizontal axis. The total numbers of particles counted as autofluorescent particles were 1.1 × 10^5^ particles/mL at 185 + 254 nm, 1.1 × 10^5^ particles/mL at 254 nm and 1.7 × 10^3^ particles/mL without deep UV irradiation. These results indicate that excitation of intracellular flavins and enhancing autofluorescence intensity by deep UV irradiation is essential for counting bacterial cells, and that the effect at 185 + 254 nm and 254 nm was almost the same. Deep UV irradiation at 185 nm in addition to 254 nm reduced the number of particles emitting strong autofluorescence and changed the distribution of the scattergram, but there was no significant difference in the counting results. Based on these results, we decided to set up a deep UV irradiation device at 185 + 254 nm just before measurement for bacterial cell counting in the PWD water. Using this protocol, we counted the number of bacteria in four commercially available bottled waters: two of the four samples were natural mineral waters that had not been sterilized, and the other two were natural mineral waters that had been sterilized by filtration or heat treatment. The bacterial counts in the two non-sterile waters were 1.1 × 10^5^ and 1.0 × 10^5^ cells/mL, and the numbers were close to the result of microscopic counting by DAPI staining (9.9 × 10^4^ and 1.2 × 10^5^ cells/mL, respectively). On the other hand, the bacterial numbers in the sterilized sample waters were 2.4 × 10^2^ and 2.0 × 10^2^ cells/mL (see Supplementary Table 3 and Supplementary Figs. 2–5).

### Bacterial cell count in the PWD water by a biofluorescent particle counter

**Figure 3:**
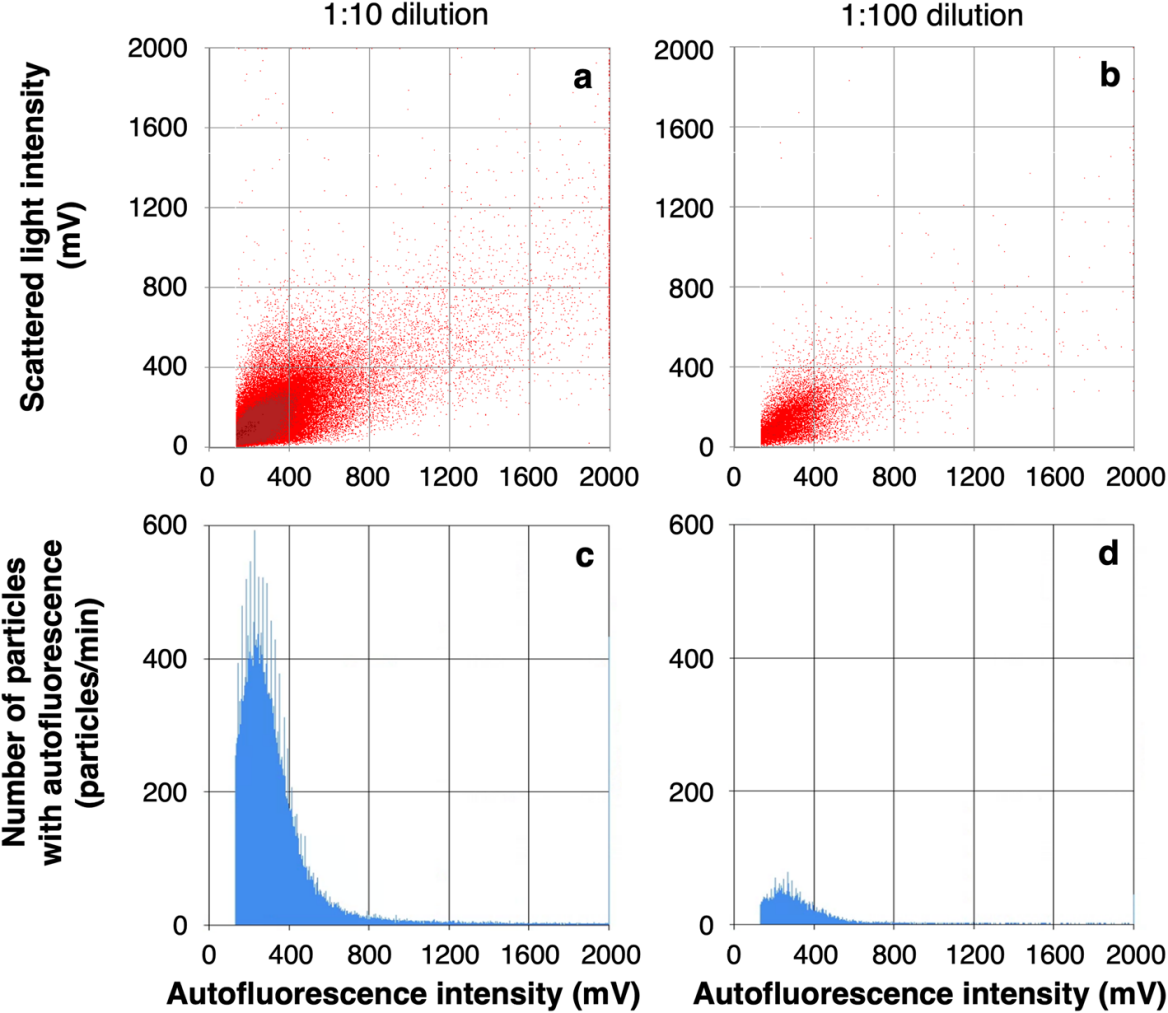
Bacterial count in the PWD water after 1:10 dilution (**a**, **c**) and 1:100 dilution (**b**, **d**) by a biofluorescent particle counter with deep UV irradiation at 185 + 254 nm. Horizontal axes indicate the flavin-derived autofluorescence intensity of the measured particles. The scattergrams (**a**, **b**) show the scattered light intensity on the vertical axis. The histograms (**c**, **d**) show the number of particles with autofluorescence intensity exceeding 133 mV on the vertical axis. The total numbers of particles counted as autofluorescent particles were the cumulative total of the number of particles on the vertical axis from 133 to 1200 mV on the horizontal axis, 1.1 × 10^5^ particles/mL (**c**) and 1.1 × 10^5^ particles/mL (**d**) after correcting for dilution factors.

Bacterial cells present in water were counted by using the developed cell counting protocol. To measure the number of bacterial cells in the PWD water by the biofluorescent particle counter, the PWD water was diluted 1:10 and 1:100 with ultrapure water before measurement (Fig. 3). Almost all particles showed autofluorescence intensities below 1200 mV (Figs. 3a, b) and the total number of particles integrated into the histograms (Fig. 3c, d) was considered to be almost equal to the number of bacteria, indicating that the biofluorescent particle counter could measure the number of bacterial cells based only on the autofluorescence intensity derived from intracellular flavin.

The number of bacterial particles present in the 1:10 and 1:100 dilutions of PWD water was measured after irradiation at 185 + 254 nm. The numbers of particles in both dilutions were correlated, and the concentration of particles in the PWD water before dilution was estimated to be 1.1 × 10^5^ particles/mL (Fig. 3). These numbers were close to the result of microscopic counting by DAPI staining, 8.2 × 10^4^ cells/mL. On the other hand, the number of bacterial cells with autofluorescence intensity above the threshold value decreased by more than 50-fold without 185 + 254 nm irradiation (data not shown).

### Bacterial community structure in the PWD water

**Figure 4:**
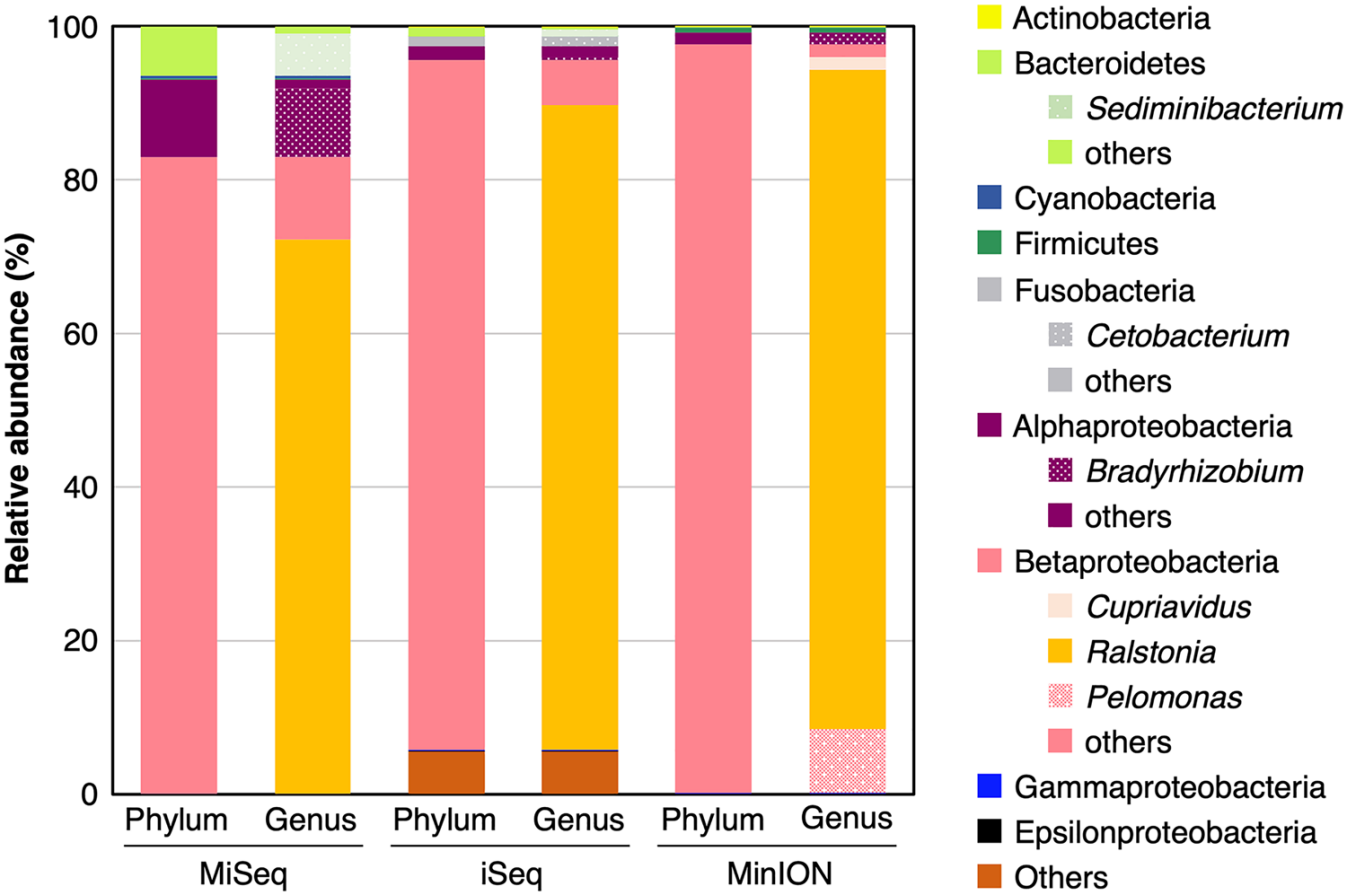
Bacterial community structure in the PWD water determined by 16S rRNA gene amplicons sequenced on Illumina MiSeq, iSeq, or Nanopore MinION.

Bacterial community structure was analyzed by ONT MinION and Illumina MiSeq and iSeq platforms. The MinION sequencing and the subsequent FASTQ 16S workflow generated 15,755 reads, among which 15,707 reads were successfully classified to the species level. The MiSeq and iSeq sequencing generated 56,038 and 440,233 merged reads after filtering and quality control, among which 56,035 and 415,558 reads were assigned at least to the phylum level, respectively. The dominant bacteria in both the phylum and genus level was Betaproteobacteria *Ralstonia* spp., consistent among MinION, MiSeq, and iSeq platforms (Fig. 4). Only the MinION platform successfully identified the predominant bacterial contaminants to the species level (Table 1), i.e., *Ralstonia pickettii*, which accounted for more than 80% of the community.

**Table 1:** Relative abundance of bacterial species in the PWD water determined by 16S rRNA gene targeted amplicon sequencing with the MinION sequencer. Genus and species with an abundance >1% are presented.

Phylum	Genus	Read count	Relative abundance (%)
	Species		
Betaproteobacteria	*Ralstonia*	13,470	85.8
	* Ralstonia pickettii*	13,261	84.4
	*Pelomonas*	1,318	8.4
	* Pelomonas saccharophila*	1,299	8.3
	*Cupriavidus*	265	1.7
Alphaproteobacteria	*Bradyrhizobium*	196	1.2

## Discussion

A self-sufficient water reclamation supply system is fundamental to the sustainable operation of very long spaceflights, where the control of microbes is the key to the safety of potable water. Historically, the abundance of bacteria has been used as an indicator of bacterial pollution in various contexts, e.g., for the safety evaluation of potable water, swimming pools, and food and the quality control of pharmaceutical manufacturing practices^16,17^. The abundance of total bacteria is significant but not sufficient to estimate the potential risk posed by bacterial contaminants because it does not provide the properties of bacterial communities, such as dominant species and species composition. Thus, simultaneous monitoring of bacterial community structure and abundance is strongly needed. Recent advancements in molecular-based microbial analysis facilitate faster and more accurate microbial diagnosis than conventional culture methods. Introducing modern techniques will be essential to developing a practical, functioning microbial surveillance system for space use.

Progress in microbial monitoring research in manned space facilities has been remarkable, with a shift from traditional culture methods to rapid microbiological methods. Nucleic acid amplification methods (loop-mediated isothermal amplification, quantitative PCR, high-throughput sequencing) and ATP-metry and flow cytometry methods have been developed^17^. In the present study, we demonstrated the high potential of the flow cytometry-based counting protocol targeting cellular flavin autofluorescence as a rapid tool for monitoring quantitative changes in bacterial bioburden. By irradiating with deep UV at both 185 nm and 254 nm just before the measurement, autofluorescence signals from dissolved organic carbons disappeared. The number of bacterial cells was compatible with the result of microscopic counting. Our protocol does not require a cell staining procedure and may be easily implemented as a continuous and routine surveillance system. At present, mercury lamps are restricted on the ISS for safety reasons, but the development of UV LEDs of sufficient intensity may make it possible to use our counting protocol on spacecraft. Continuous monitoring of the number of bacterial cells allows real-time detection of changes in bacterial bioburden, leading to early alert of bacterial outbreaks in potable water.

We also demonstrated the efficacy of the MinION nanopore sequencer in rapidly characterizing bacterial community structure, which result was in good agreement with the results from the Illumina MiSeq and iSeq platforms. The MinION platform has low installation costs due to its small size and shorten sequencing time because it does not require DNA synthesizing during sequencing^12,13^. In addition, although the sequencing accuracy of nanopore sequencers is still lower than that of short-read (up to 600 bp), high-throughput sequencers such as Illumina MiSeq, the MinION platform would have higher probabilities in identifying bacterial taxa to the species level based on long-read seqencing^18,19^. Here, we targeted the whole 16S rRNA gene (c. 1.5 kbp) in the MinION platform and the 16S rRNA gene V4 region (c. 292 bp) in the MiSeq and iSeq platforms. The MinION was the only platform that successfully identified the dominant bacteria to the species level as *R. pickettii*, which was consistent with the identification of bacterial isolates with the MALDI TOF–MS. Because of the high error rates of the MinION platform, it requires higher read depth^20^ to achieve species-level identification. Thus, the taxonomic resolution of the MinION may decrease in highly diverse bacterial communities. However, the present study showed that the MinION platform would exhibit high taxonomic resolution with moderate sequencing depth, i.e., 15,755 total reads in this study, when a few species are dominant, which would often be the case with outbreaks of particular species or strain.

The future direction of microbial surveillance will be the automation of real-time monitoring systems in manned space facilities. A portable semi-quantitative PCR instrument called RAZOR EX (BioFire Defense, Salt Lake City, USA) was tested aboard the ISS in 2016–2017 and proven sufficiently accurate for the quantitative detection of a target DNA in water^21^. Urbaniak et al.^22^ recently demonstrated the efficacy of an automated water concentrator, the ISS Smart Sample Concentrator (iSSC), for its future use in monitoring low biomass water samples on the ISS. Both studies stressed their semi-automated workflow, which would ease the crew time required for onboard microbial monitoring. In this study, we proposed direct counting method of bacteria using a biofluorescent particle counter. Our method enables rapid counting of bacterial cells in water by directly injecting water samples into the particle counter. If bacterial concentration becomes excessive, it can be easily solved by diluting the sample, which does not require any specific resources, including crew time. As to the on-board microbial community analysis, the "swab-to-sequencer method" proposed by Stahl-Rommel et al.^23^ and on-board nucleic acid extraction protocols from water^11^ can be combined to develop a direct evaluation method of bacterial community structure in water.​​​​

Shortly, long-duration manned space missions, such as the lunar orbital platform Gateway, the moon base, or a manned planetary spacecraft, will require potable water recycling and supply systems that do not rely on transporting water from the earth. We will continue to further improve and automate our proposed protocols for practical use in manned space facilities to ensure the hygienic and microbiological safety and the stable supply of purified potable water there, which is a more complex and essential task than ever before.

## Materials and methods

### PWD water sampling in the ISS

On January 8, 2021, an astronaut collected 350 mL of potable water from the PWD on the United States On-orbit Segment (USOS) of the ISS into NASA’s Post-Flight Analysis Bag. It is made of fluorinated ethylene propylene (FEP) with polypropylene female Luer lock ports and is used for water sample collection and analyses by NASA. The ISS Potable Water Sampling Bag was loaded onto the SpaceX CRS-21 (SpX-21) cargo dragon capsule, which splashed down in the Gulf of Mexico on January 14, 2021 and was transported to NASA's Kennedy Space Center (KSC) at room temperature. After arrival at the KSC, the bag was stored at 4 °C and transported from the KSC to the Tsukuba Space Center, JAXA, Japan, arriving on January 21, 2021. Ground control water was prepared in our laboratory by collecting ultrapure water in the same bag at the same time as the PWD water sampling and was stored under the same conditions until the measurement.

### Total direct counting and CFU counting

Bacteria present in the PWD water were filtered through a black polycarbonate filter (pore size of 0.2 µm, ADVANTEC). Filters were rinsed twice with bacterium-free distilled water. Then, 1 µg/ml of DAPI in distilled water or 150 µg/ml of 6-CFDA in phosphate buffer (0.3 M phosphate (pH 8.5), 15% NaCl, 1.5 mM EDTA) was applied to the filters and incubated for 3 min at room temperature under dark conditions. Filters were rinsed twice, mounted on glass microscope slides with non-fluorescent immersion oil, and examined using an epifluorescence microscope (DM2500, Leica Microsystems) with an oil immersion objective. The number of CFU was determined by spreading diluted ISS potable water on BD TSA and BD BBL R2A agar (Becton, Dickinson and Company), which were incubated for one week before counting at 30 °C and 22 °C, respectively.

### MALDI-TOF MS spectrometry for identification of isolated bacteria

A MALDI-TOF MS was used for direct identification of bacteria grown on TSA and R2A agar^24^. 18 and 20 colonies isolated from TSA and R2A were smeared onto the MALDI MSP 96 polished steel target plate. One microliter of 70% formic acid was deposited onto each sample spot and allowed to dry. Then, 1 µL of matrix solution (α-cyano-4-hydroxycinnamic acid (Bruker Daltonics) dissolved in 50% acetonitrile, 47.5% water, and 2.5% trifluoroacetic acid) was applied onto each sample spot and allowed to dry. Bruker Bacterial Test Standard, *Escherichia coli* DH5α was used for calibration. Measurements were performed with a Microflex mass spectrometer (Bruker Daltonics) using the flexControl software version 3.4. Mass spectra were acquired in a linear positive extraction mode ranging from 2000 to 20,000 Da. The spectra were analyzed using the MALDI Biotyper 3.1 software with Bruker library BDAL Ver. 6 and Filamentous Fungi Library Ver. 1.0 (Bruker Daltonics). A manufacturer-recommended cutoff score was used for identification (Supplementary Table 2).

### Bacterial cell counting using a biofluorescent particle counter

A commercially available biofluorescent particle counter (XL-10BT1, Rion Co. Ltd.) was used in this study. It had two detectors. One was a photodiode for measuring the scattered light intensity, which indicates particle size. The other was a photomultiplier tube for measuring the autofluorescence intensity, which is an indicator of the physiological activity of bacteria. This allowed the scattered light intensity and the autofluorescence intensity to be measured simultaneously.

We developed a protocol to measure bacterial cells without staining using the biofluorescent particle counter, identifying particles emitting autofluorescence from flavin and counting them as bacterial cells. Flavin is a ubiquitous pigment in bacterial cells, which emits autofluorescence at 510 nm when irradiated with 405 nm excitation light^25^. The amount of intracellular flavin is closely related to the physiological activity of bacteria^25^, and thus bacteria with low physiological activity may not be detected due to low autofluorescence intensities. To solve this problem, we irradiated particles with deep UV irradiation, at a wavelength of 254 nm or two wavelengths of 185 nm and 254 nm, to oxidize flavin and enhance autofluorescence intensity immediately before the measurement under irradiation at 405 nm, which was carried out using a deep UV irradiation device (XL-28A, RION Co. Ltd.) equipped with a low-pressure mercury lamp. Since deep UV irradiation is known to degrade organic carbon^26,27^, the deep UV irradiation incorporated in our bacterial cell counting protocol was also used for reducing signals from dissolved organic carbon in the PWD water. Particle counting was performed at a flow rate of 10 mL/min and a measurement time of 60 s. Deep UV irradiation dose was over 1000 mJ/cm^2^. Before measuring bacterial cells, ultrapure water was used to set a cutoff threshold for electrical noise derived from the biofluorescent particle counter, which was determined as 133 mV. To exclude particles with extremely high autofluorescence intensity from being counted as bacteria, upper threshold was defined at 1200 mV when counting bacterial cells.

### DNA extraction

Bacterial cells present in 50 mL of the PWD water were trapped onto an autoclaved polycarbonate membrane filter (pore size of 0.2 μm; Advantec, Tokyo, Japan). Bacterial DNA was extracted with the method described in Ichijo et al*.*^2^. The DNA was finally eluted with 50 µL of TE buffer.

### Amplicon sequencing of bacterial 16S rRNA gene by ONT MinION platform

Amplicon sequencing targeting the full length of the 16S rRNA gene was performed using MinION equipped with R9.4.1 flow cell (Oxford Nanopore Technologies, Oxford, UK). A 16S rRNA sequencing library was constructed from 10 µL of extracted DNA using the 16S barcoding kit (Oxford Nanopore Technologies). The library construction was performed according to the manufacturer's instructions except that DNA amplification was carried out using KAPA HiFi HotStart ReadyMix (KAPA Biosystems, MA, USA) with the following thermal cycling conditions: 2 min at 95 °C, 25 cycles of 20 s at 98 °C, 30 s at 60 °C, and 2 min at 72 °C, and 5 min at 72 °C. Sequencing was carried out with Oxford Nanopore's MinKNOW software and basecalls were performed using Guppy (v. 4.3.4) in fast mode using the config file dna_r9.4.1_450bps_fast.cfg. Generated FASTQ files were further analyzed for taxonomic classification using the cloud-based EPI2ME FASTQ 16S workflow with a quality score ≥ 7 for quality filtering.

### Amplicon sequencing of bacterial 16S rRNA gene by Illumina MiSeq and iSeq platforms

Amplicon sequencing targeting the 16S rRNA gene V4 region was performed using the 300-bp paired-end MiSeq platform with MiSeq Reagent Kit v2 and 150-bp paired-end iSeq platform with iSeq 100 i1 Reagent (Illumina, CA, USA). A two-step PCR was performed to construct paired-end libraries. In the first PCR, the V4 region of the prokaryotic 16S rRNA gene was amplified from the DNA sample using F515 and R806 primers^28^ with the Illumina overhang adapters. The first PCR reactions were carried out in triplicate in 12 µL reaction volume containing 3 µL of the template and 0.6 µM each of forward and reverse primers in 1 × KAPA HiFi HotStart ReadyMix (KAPA Biosystems) using thermal cycling of 2 min at 95 °C, 35 cycles of 20 s at 98 °C, 15 s at 60 °C, and 30 s at 72 °C, and 5 min at 72 °C. The triplicate PCR products were pooled and purified using Agencourt AMPure XP (Beckman Coulter, CA, USA) according to the manufacturer's instructions. A second PCR (12 cycles) was performed to attach dual indices and Illumina sequencing adapters to the purified first PCR products using Nextera XT Index Kit v2 Set C (Illumina). Finally, the indexed amplicons were purified by electrophoresis using E-Gel SizeSelect II Agarose Gels (Thermo Fisher Scientific, MA, USA). The DNA concentrations of the indexed amplicons were quantified by a Qubit 4 Fluorometer using dsDNA HS Assay Kit (Thermo Fisher Scientific) and pooled in equal amounts for library construction. The library was diluted to 1 nM and spiked with 20% PhiX Control v3 (Illumina), then diluted to 50 pM, loaded into MiSeq and iSeq cartridges, and sequenced according to the manufacturer's instructions.

FASTQ files generated from MiSeq and iSeq sequencing were analyzed separately using QIIME 2 pipeline^29^ (v. 2021.8). Denoising sequences, merging paired-end reads, and chimera filtering were performed using the qiime dada2 denoise-paired method of the DADA2^30,31^ plugin in QIIME 2, where parameters were adjusted from defaults both for MiSeq and iSeq data as follows: –p-trim-left-f 19, –p-trim-left-r 20, –p-max-ee-f 1.0, and –p-max-ee-r 1.0. In addition to these adjustments, the parameters –p-trunc-len-f and –p-trunc-len-r were adjusted to 263 and 226 for the MiSeq data to truncate forward and reverse reads at the position with a median quality score lower than 30. As to the iSeq data, forward and reverse reads were not truncated. The parameter –p-min-overlap, which controls the minimum overlap required for merging the paired reads, was reduced from the default (12) to 5 to enable merging. The yielded non-chimeric sequences, called Amplicon Sequence Variants (ASVs), were assigned to taxonomic groups using ‘qiime feature-classifier classify-sklearn’^31^ using a naïve Bayes classifier pre-trained on the Silva 138 99% database^32^ for 515F/806R region of 16S rRNA (silva-138-99-515-806-nb-classifier.qza) with a confidence threshold of 0.985.

## Supplementary Information

Supplementary Information.

## Data Availability

The data that support the findings of this study are available from the corresponding author (MN) upon reasonable request. Bacterial DNA sequences were deposited in the DDBJ sequence read archive (DRA) under accession number of DRA014205.

## References

[1] Checinska Sielaff A (2019). Characterization of the total and viable bacterial and fungal communities associated with the International Space Station surfaces. Microbiome.

[2] Ichijo T, Yamaguchi N, Tanigaki F, Shirakawa M, Nasu M (2016). Microbiomes of the dust particles collected from the International Space Station and Spacecraft Assembly Facilities. NPJ Microgravity.

[3] Checinska A (2015). Microbiomes of the dust particles collected from the International Space Station and Spacecraft Assembly Facilities. Microbiome.

[4] O’Rourke A, Lee MD, Nierman WC, Craig Everroad R, Dupont CL (2020). Genomic and phenotypic characterization of *Burkholderia* isolates from the potable water system of the International Space Station. PLoS ONE.

[5] Wong WC, Dudinsky LA, Garcia VM, Ott CM, Castro VA (2010). Efficacy of various chemical disinfectants on biofilms formed in spacecraft potable water system components. Biofouling.

[6] Toon, K. P. & Lovell, R. W. International space station United States on-orbit segment potable water dispenser on-orbit functionality vs. design. in *40th International Conference on Environmental Systems* (2010).

[7] Bruce RJ, Ott CM, Skuratov VM, Pierson DL (2005). Microbial surveillance of potable water sources of the international space station. SAE Tech. Pap..

[8] Maryatt, B. W. & Smith, M. J. Microbial growth control in the international space station potable water dispenser. in *47th International Conference on Environmental Systems* ICES-2017–231 (2017).

[9] Straub II, J. E. *et al.* Chemical characterization of ISS potable water collected in 2017. In *48th International Conference on Environmental Systems* ICES-2018-282 (2018).

[10] Novikova N (2006). Survey of environmental biocontamination on board the International Space Station. Res. Microbiol..

[11] Amalfitano, S. *et al.* Water and microbial monitoring technologies towards the near future space exploration. *Water Res.* **177**. 10.1016/j.watres.2020.115787 (2020). 10.1016/j.watres.2020.115787 32315899

[12] Kono N, Arakawa K (2019). Nanopore sequencing: Review of potential applications in functional genomics. Dev. Growth Differ..

[13] van Dijk EL, Jaszczyszyn Y, Naquin D, Thermes C (2018). The third revolution in sequencing technology. Trends Genet..

[14] Burton AS (2020). Off earth identification of bacterial populations using 16S rDNA nanopore sequencing. Genes.

[15] Castro-Wallace SL (2017). Nanopore DNA sequencing and genome assembly on the International Space Station. Sci. Rep..

[16] Gad, S. C. *Pharmaceutical Manufacturing Handbook: Production and Processes*. *Pharmaceutical Manufacturing Handbook: Production and Processes* (2007). 10.1002/9780470259818

[17] Amalfitano S (2018). Water quality and total microbial load: A double-threshold identification procedure intended for space applications. Front. Microbiol..

[18] Benítez-Páez A, Portune KJ, Sanz y (2016). Species-level resolution of 16S rRNA gene amplicons sequenced through the MinION^TM^ portable nanopore sequencer. Gigascience.

[19] Nygaard AB, Tunsjø HS, Meisal R, Charnock C (2020). A preliminary study on the potential of Nanopore MinION and Illumina MiSeq 16S rRNA gene sequencing to characterize building-dust microbiomes. Sci. Rep..

[20] Petersen LM, Martin IW, Moschetti WE, Kershaw CM, Tsongalis GJ (2020). Third-generation sequencing in the clinical laboratory: Exploring the advantages and challenges of nanopore sequencing. J. Clin. Microbiol..

[21] Khodadad CLM (2021). A microbial monitoring system demonstrated on the international space station provides a successful platform for detection of targeted microorganisms. Life.

[22] Urbaniak, C., Mhatre, S., Grams, T., Parker, C. & Venkateswaran, K. Validation of the international space station smart sample concentrator for microbial monitoring of low biomass water samples. *J. Biomol. Tech.* **4**. 10.7171/jbt.20-3104-005 (2020). 10.7171/jbt.20-3104-005 PMC7566613 33100920

[23] Stahl-Rommel S (2021). Real-time culture-independent microbial profiling onboard the international space station using nanopore sequencing. Genes (Basel).

[24] Schmitt BH, Cunningham SA, Dailey AL, Gustafson DR, Patel R (2013). Identification of anaerobic bacteria by Bruker Biotyper matrix-assisted laser desorption ionization-time of flight mass spectrometry with on-plate formic acid preparation. J. Clin. Microbiol..

[25] Li J‐K, Asali EC, Humphrey AE, Horvath JJ (1991). Monitoring cell concentration and activity by multiple excitation fluorometry. Biotechnol. Prog..

[26] Allard B, Borén H, Pettersson C, Zhang G (1994). Degradation of humic substances by UV irradiation. Environ. Int..

[27] Kiattisaksiri P (2011). Vacuum ultraviolet irradiation for mitigating dissolved organic nitrogen and formation of haloacetonitriles. Environ. Res..

[28] Caporaso JG (2011). Global patterns of 16S rRNA diversity at a depth of millions of sequences per sample. Proc. Natl. Acad. Sci. USA.

[29] Bolyen E (2019). Reproducible, interactive, scalable and extensible microbiome data science using QIIME 2. Nat. Biotechnol..

[30] Callahan BJ (2016). DADA2: High-resolution sample inference from Illumina amplicon data. Nat. Methods.

[31] Bokulich NA (2018). Optimizing taxonomic classification of marker-gene amplicon sequences with QIIME 2’s q2-feature-classifier plugin. Microbiome.

[32] Quast C (2013). The SILVA ribosomal RNA gene database project: Improved data processing and web-based tools. Nucleic Acids Res..

