# Supplementary material for: Bacterial bioburden and community structure of potable water used in the International Space Station
Source: Sci Rep. 2022 Sep 29;12:16282. doi: 10.1038/s41598-022-19320-3 (PMC9522912; doi:10.1038/s41598-022-19320-3)
Supplement: Supplementary file 1 — Supplementary Information. [file 41598_2022_19320_MOESM1_ESM.pdf]

Bacterial bioburden and community structure of potable water used in the International Space Station

Tomoaki Ichijo, Kimiko Uchii, Kazuma Sekimoto, Takashi Minakami, Takashi Sugita, Masao Nasu, Takashi Yamazaki

Supplementary Information

Supplementary Table1. MALDI Biotyper Classification Results

Analyte ID	Quality	Organism (best match)	Score value	Organism (second best match)	Score value
Control 1	++	*Escherichia coli*	2.095	*Escherichia coli*	2.079
Control 2	++	*Escherichia coli*	2.264	*Escherichia coli*	2.240
TSA 1	+++	*Ralstonia pickettii*	2.329	*Ralstonia pickettii*	2.306
TSA 2	+++	*Ralstonia pickettii*	2.435	*Ralstonia pickettii*	2.218
TSA 3	+++	*Ralstonia pickettii*	2.411	*Ralstonia pickettii*	2.330
TSA 4	+++	*Ralstonia pickettii*	2.399	*Ralstonia pickettii*	2.394
TSA 5	+++	*Ralstonia pickettii*	2.411	*Ralstonia pickettii*	2.369
TSA 6	+++	*Ralstonia pickettii*	2.386	*Ralstonia pickettii*	2.336
TSA 7	++	*Ralstonia pickettii*	2.288	*Ralstonia pickettii*	2.255
TSA 8	+++	*Ralstonia pickettii*	2.332	*Ralstonia pickettii*	2.194
TSA 9	++	*Ralstonia pickettii*	2.291	*Ralstonia pickettii*	2.257
TSA 10	+++	*Ralstonia pickettii*	2.407	*Ralstonia pickettii*	2.257
TSA 11	+++	*Ralstonia pickettii*	2.481	*Ralstonia pickettii*	2.446
TSA 12	+++	*Ralstonia pickettii*	2.414	*Ralstonia pickettii*	2.213
TSA 13	+++	*Ralstonia pickettii*	2.451	*Ralstonia pickettii*	2.395
TSA 14	+++	*Ralstonia pickettii*	2.373	*Ralstonia pickettii*	2.238
TSA 15	+++	*Ralstonia pickettii*	2.473	*Ralstonia pickettii*	2.411
TSA 16	+++	*Ralstonia pickettii*	2.530	*Ralstonia pickettii*	2.445
TSA 17	+++	*Ralstonia pickettii*	2.452	*Ralstonia pickettii*	2.441
TSA 18	+++	*Ralstonia pickettii*	2.429	*Ralstonia pickettii*	2.371
R2A 1	+++	*Ralstonia pickettii*	2.358	*Ralstonia pickettii*	2.301
R2A 2	+++	*Ralstonia pickettii*	2.362	*Ralstonia pickettii*	2.330
R2A 3	+++	*Ralstonia pickettii*	2.498	*Ralstonia pickettii*	2.400
R2A 4	+++	*Ralstonia pickettii*	2.349	*Ralstonia pickettii*	2.324
R2A 5	++	*Ralstonia pickettii*	2.293	*Ralstonia pickettii*	2.288
R2A 6	+++	*Ralstonia pickettii*	2.372	*Ralstonia pickettii*	2.284
R2A 7	+++	*Ralstonia pickettii*	2.364	*Ralstonia pickettii*	2.358
R2A 8	+++	*Ralstonia pickettii*	2.378	*Ralstonia pickettii*	2.207
R2A 9	+++	*Ralstonia pickettii*	2.422	*Ralstonia pickettii*	2.405
R2A 10	+++	*Ralstonia pickettii*	2.398	*Ralstonia pickettii*	2.396
R2A 11	+++	*Ralstonia pickettii*	2.301	*Ralstonia pickettii*	2.193
R2A 12	+++	*Ralstonia pickettii*	2.394	*Ralstonia pickettii*	2.239
R2A 13	+++	*Ralstonia pickettii*	2.539	*Ralstonia pickettii*	2.381
R2A 14	++	*Ralstonia pickettii*	2.052	*Ralstonia pickettii*	1.997
R2A 15	++	*Ralstonia pickettii*	2.245	*Ralstonia pickettii*	2.220
R2A 16	++	*Ralstonia pickettii*	2.091	*Ralstonia pickettii*	2.069
R2A 17	+++	*Ralstonia pickettii*	2.438	*Ralstonia pickettii*	2.408
R2A 18	+++	*Ralstonia pickettii*	2.438	*Ralstonia pickettii*	2.322
R2A 19	+++	*Ralstonia pickettii*	2.459	*Ralstonia pickettii*	2.349
R2A 20	-	no peaks found	<0	no peaks found	<0

Supplementary Table2. Meaning of score value

Range	Description	Symbols
2.300…3.000	highly probable species identification	(+++)
2.000…2.299	secure genus identification, probable species identification	(++)
1.700…1.999	probable genus identification	(+)
0.000…1.699	probable genus identification	(-)

Supplementary Table3. Bacterial numbers in commercially available natural mineral waters.

		BPC[a] (cells/mL)	TDC[b] (cells/mL)	TSA plate counts[c] (CFU/mL)	R2A plate counts[d] (CFU/mL)
Natural mineral water A	non-sterile	1.1×10^{5}	9.9×10^{4}	< 300	7.3×10^{3}
Natural mineral water B	non-sterile	1.0×10^{5}	1.2×10^{5}	< 300	6.2×10^{2}
Natural mineral water C	sterile	2.4×10^{2}	$< 8.4\times10^{2}$ [e]	< 300	< 300
Natural mineral water D	sterile	2.0×10^{2}	$< 8.4\times10^{2}$ [e]	< 300	< 300

a, biofluorescent particle counter; *b*, total direct counting using DAPI
c, Incubated on TSA medium at 30˚C for 7 days
d, Incubated on R2A medium at 22˚C for 7 days
e, under quantification limit (< 2 cells/microscopic field)

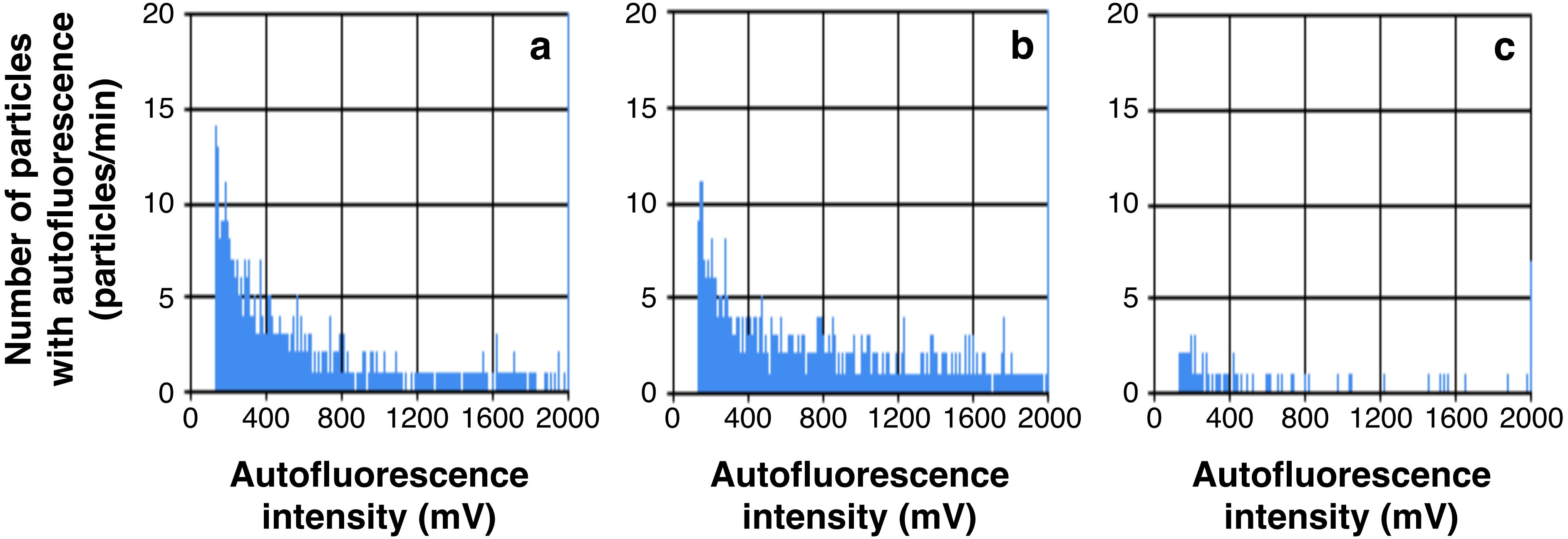

Supplementary Fig. 1

Particle count with autofluorescence by a biofluorescent particle counter with deep UV irradiation at 185 nm +254 nm in the PWD water passed through a 0.2 µm filter (a) , the ground control water (b) and the ultrapure water using for the ground control before it was sealed in the ISS Potable Water Sampling Bag (c). The total numbers of particles counted as autofluorescence particles were the cumulative total of the number of particles on the vertical axis from 133 mV to 1200 mV on the horizontal axis, 1.6×10^2 particles/mL (a) , 1.3×10^2 particles/mL (b) and 8.4 particles/mL (c).

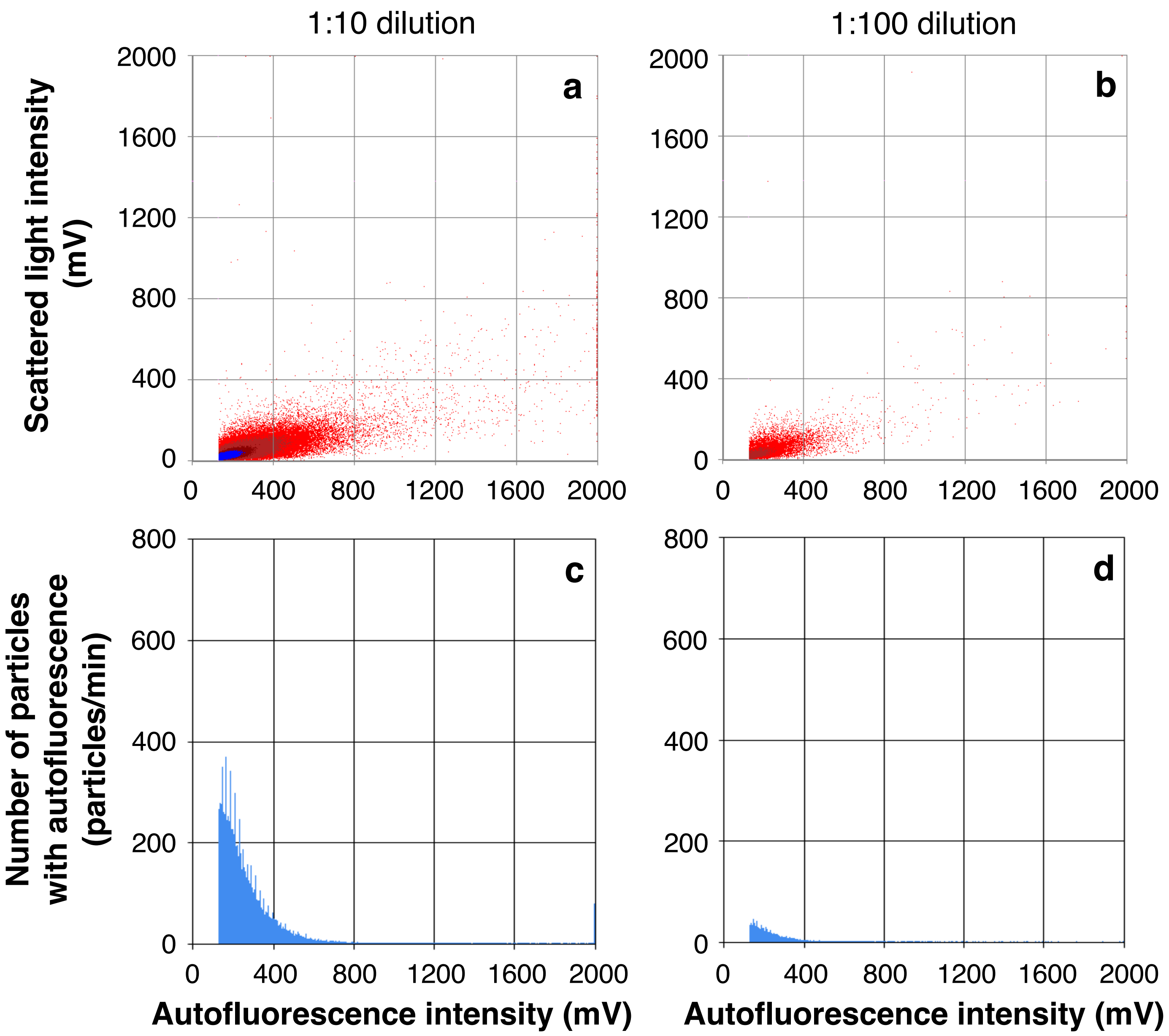

Supplementary Fig. 2
Bacterial count in a commercial available natural mineral water (non-sterile; natural mineral water A in supplementary Table 3) after 1:10 dilution (a, c) and 1:100 dilution (b, d) by a biofluorescent particle counter with deep UV irradiation at 185 nm + 254 nm. Horizontal axes indicate the flavin-derived autofluorescence intensity of the measured particles. The scattergrams (a, b) show the scattered light intensity on the vertical axis. The histograms (c, d) show the number of particles with autofluorescence intensity exceeding 133 mV on the vertical axis. The total numbers of particles counted as autofluorescence particles were the cumulative total of the number of particles on the vertical axis from 133 mV to 1200 mV on the horizontal axis, 1.1×10^4 particles/mL (c) and 1.0×10^3 particles/mL (d).

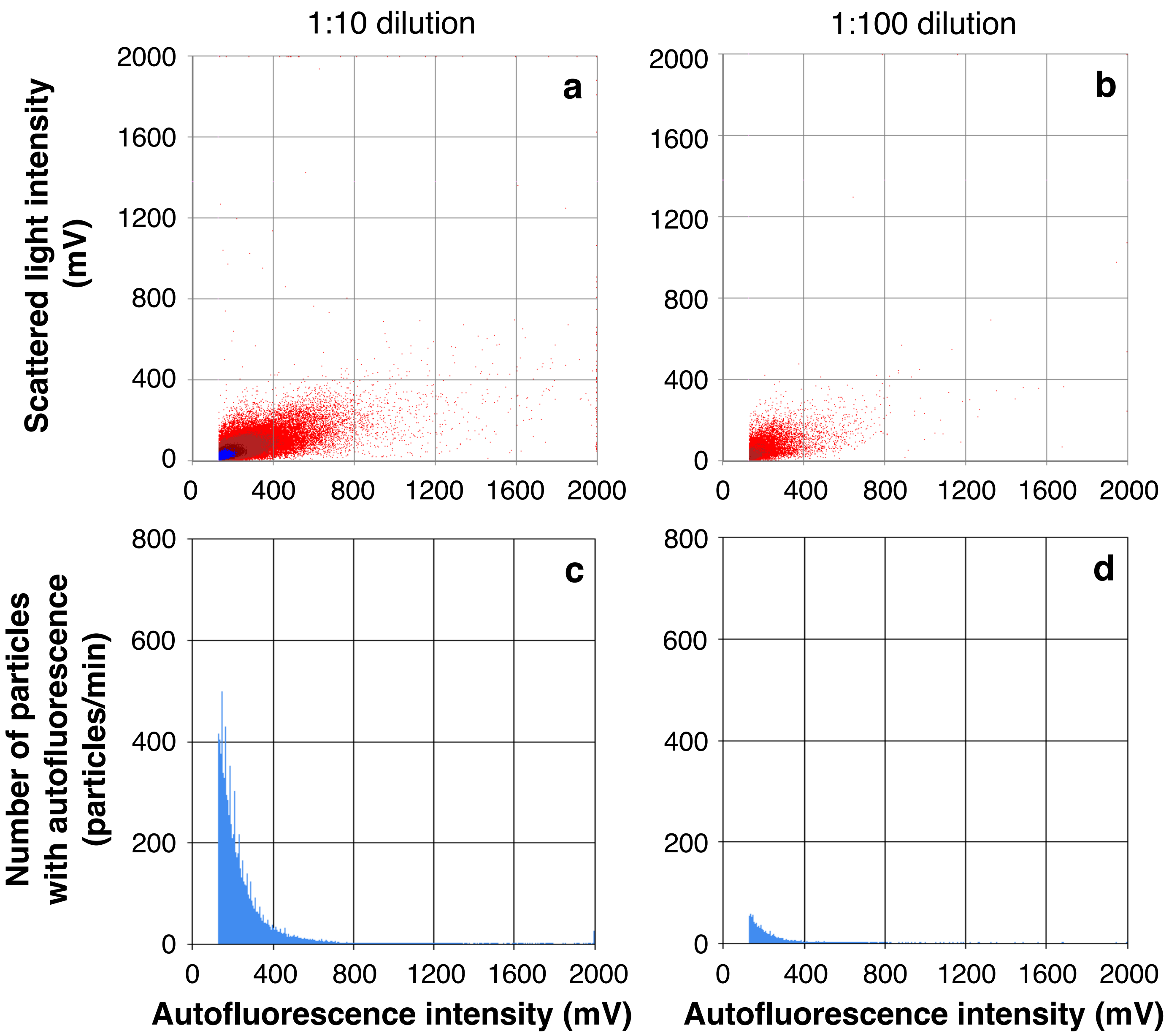

Supplementary Fig. 3
Bacterial count in a commercial available natural mineral water (non-sterile; natural mineral water B in supplementary Table 3) after 1:10 dilution (a, c) and 1:100 dilution (b, d) by a biofluorescent particle counter with deep UV irradiation at 185 nm + 254 nm. Horizontal axes indicate the flavin-derived autofluorescence intensity of the measured particles. The scattergrams (a, b) show the scattered light intensity on the vertical axis. The histograms (c, d) show the number of particles with autofluorescence intensity exceeding 133 mV on the vertical axis. The total numbers of particles counted as autofluorescence particles were the cumulative total of the number of particles on the vertical axis from 133 mV to 1200 mV on the horizontal axis, 1.0×10^4 particles/mL (c) and 1.0×10^3 particles/mL (d).

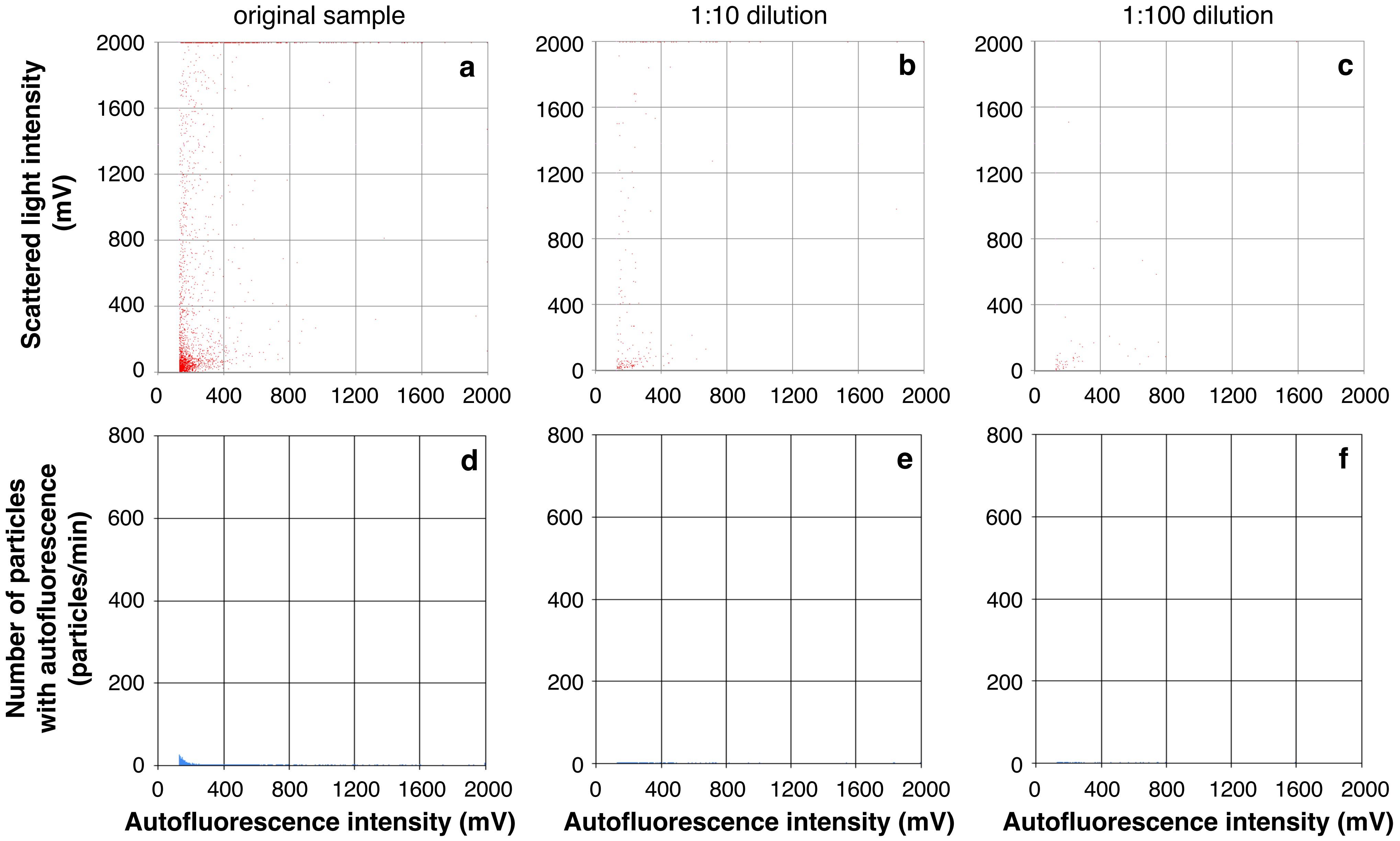

Supplementary Fig. 4

Bacterial count in a commercial available natural mineral water (sterile; natural mineral water C in supplementary Table 3) (a, d) after 1:10 dilution (b, e) and 1:100 dilution (c, f) by a biofluorescent particle counter with deep UV irradiation at 185 nm + 254 nm. Horizontal axes indicate the flavin-derived autofluorescence intensity of the measured particles. The scattergrams (a, b, c) show the scattered light intensity on the vertical axis. The histograms (d, e, f) show the number of particles with autofluorescence intensity exceeding 133 mV on the vertical axis. The total numbers of particles counted as autofluorescence particles were the cumulative total of the number of particles on the vertical axis from 133 mV to 1200 mV on the horizontal axis, 2.4×10^2 particles/mL (d), 2.2×10^1 particles/mL (e) and 4.9 particles/mL (f).

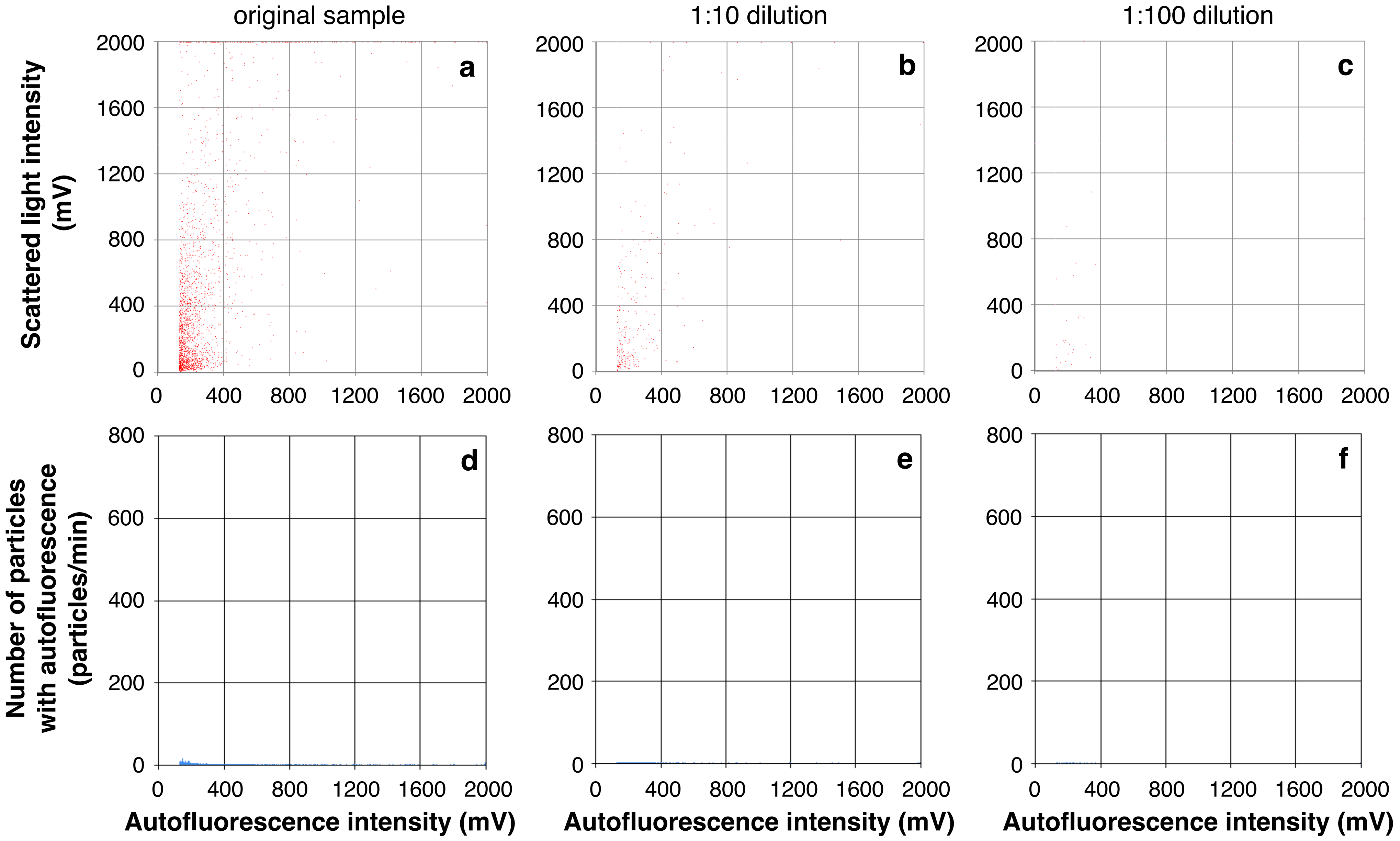

Supplementary Fig. 5
Bacterial count in a commercial available natural mineral water (sterile; natural mineral water D in supplementary Table 3) (a, d) after 1:10 dilution (b, e) and 1:100 dilution (c, f) by a biofluorescent particle counter with deep UV irradiation at 185 nm + 254 nm. Horizontal axes indicate the flavin-derived autofluorescence intensity of the measured particles. The scattergrams (a, b, c) show the scattered light intensity on the vertical axis. The histograms (d, e, f) show the number of particles with autofluorescence intensity exceeding 133 mV on the vertical axis. The total numbers of particles counted as autofluorescence particles were the cumulative total of the number of particles on the vertical axis from 133 mV to 1200 mV on the horizontal axis, 2.0×10^2 particles/mL (d), 2.4×10^1 particles/mL (e) and 3.0 particles/mL (f).
